# Mayfly *Ephemeraglaucops* (Ephemeroptera, Ephemeridae) recorded in the Czech Republic after almost a century

**DOI:** 10.3897/BDJ.10.e90950

**Published:** 2022-09-09

**Authors:** Pavel Sroka, Jindřiška Bojková, Vojtech Kolar

**Affiliations:** 1 Biology Centre of the Czech Academy of Sciences, Institute of Entomology, České Budějovice, Czech Republic Biology Centre of the Czech Academy of Sciences, Institute of Entomology České Budějovice Czech Republic; 2 Department of Botany and Zoology, Faculty of Science, Masaryk University, Brno, Czech Republic Department of Botany and Zoology, Faculty of Science, Masaryk University Brno Czech Republic; 3 Department of Ecosystem Biology, Faculty of Science, University of South Bohemia, České Budějovice, Czech Republic Department of Ecosystem Biology, Faculty of Science, University of South Bohemia České Budějovice Czech Republic

**Keywords:** aquatic insects, distribution, faunistics, mayflies

## Abstract

**Background:**

The mayfly Ephemera (Sinephemera) glaucops Pictet, 1843 has been considered regionally extinct in the Czech Republic, with the last occurrence dating from 1933. Its extinction was connected with the anthropogenic changes of lowland rivers, forming the original habitat of *E.glaucops* within the area of the Czech Republic. However, the species has been reported as spreading in man-made, often post-industrial waterbodies in Germany, The Netherlands and Austria since the 1970s.

**New information:**

We report *E.glaucops* from the Czech Republic, based on 27 larvae collected in the North Bohemia lignite basin in 2018. All individuals were found at one locality – a small kaolin pit in the shallow part near the shore, mostly without macrophytes. We provide details about the locality and morphological diagnostic characters of *E.glaucops*. This study highlights the importance of post-industrial sites for aquatic biodiversity as surrogate biotopes for degraded original habitats.

## Introduction

The mayfly (Ephemeroptera) assemblages have been affected by profound anthropogenic changes of freshwater habitats over the last century, which often resulted in local or regional species loss (e.g. Malmquist and Rundle 2002). Human activities concentrated mainly on large lowland rivers and their floodplains, with local fauna suffering the most significant changes in species composition and local extinctions (Soldán et al. 2017). One of the species originally inhabiting large rivers in the Czech Republic was *Ephemeraglaucops* (family Ephemeridae). It disappeared from the territory of the Czech Republic early in the 20^th^ century, with the finding from the Elbe River published by Pawlik (1933) being the last reliable record (Soldán et al. 1998, Soldán et al. 2017). This species is listed as regionally extinct in the current Red List of Threatened Species of the Czech Republic (Soldán et al. 2017).

Geographically, the species is relatively widespread; its distributional range covers large part of Europe from Sweden in the north to Spain and North Africa in the south-west and Ukraine, Romania, Serbia and possibly Greece in the south-east. The type locality is specified as Genthod, near Geneva, based on four syntypes deposited in the Natural History Museum of Geneva (Sartori and Bauernfeind 2020). Nevertheless, *E.glaucops* is generally rare with fragmentary distribution (Bauernfeind and Soldán 2012). It has been recorded from epipotamal rivers, oligotrophic or mesotrophic lakes with gravel substrate (such as Lac de Genève, Lac d'Annecy, Lago di Garda, Lac des Quatre-Cantons and Lac de Constance), but also from different man-made waterbodies (Jacob et al. 1975, Studemann et al. 1992). In Central Europe, its recent focal habitats are man-made oligotrophic waterbodies, especially originating from different types of surface mining (e.g. Jacob et al. 1975, Braasch and Mey 1977, Haybach and Fischer 1994, Hutter and Graf 1994, Zahn 2003, Koese 2008).

Apart from *E.glaucops*, three species of the genus *Ephemera* have been recorded in the territory of the Czech Republic, namely Ephemera (Ephemera) danica Müller, 1764, Ephemera (Ephemera) lineata Eaton, 1870 and Ephemera (Ephemera) vulgata Linnaeus, 1758. Two of these species, *E.vulgata* and *E.danica* are common, whereas *E.lineata* is very rare and considered endangered (Zahrádková et al. 2009, Soldán et al. 2017).

Recently, we conducted an extensive survey of freshwater invertebrates in northern and eastern Bohemia and identified *E.glaucops* in one of the localities studied. The goal of the present paper is to report on the occurrence of *E.glaucops* in the Czech Republic, to provide characteristics of its habitat and to summarise the diagnostic characters of *E.glaucops* from other *Ephemera* species occurring in the Czech Republic.

## Materials and methods

In 2018–2019, we surveyed altogether 20 different types of freshwater post-industrial sites, such as mining subsidence, sandpits, ash lagoons, quarries and kaolin pits in northern and eastern Bohemia. The localities were visited three times (in summer, autumn and spring). At each locality, we selected three sites of different microhabitats and sampled them by standardised methods: box trap and time standardised (5 min/site) sweeping with a kitchen strainer to maximise species capture (Klečka and Boukal 2014). We also measured physico-chemical parameters of water with a portable YSI multimeter (type 556 MPS), substrate pH with Eutech probe, equipped with an Orion 9103BNWP electrode and water transparency with the Sneller tube.

## Taxon treatments

### Ephemera (Sinephemera) glaucops

Pictet, 1843

AAAC5765-3094-52DD-937C-9B8F04CE7ADF

Ephemera glaucops Pictet, 1843; Hist. nat. gen. part. Ins. Névropt., p. 132
Ephemera (Sinephemera) glaucops Pictet, 1843 in Kluge (2004)
Ephemera (Sinephemera) glaucops Pictet, 1843 in Bauernfeind and Soldán (2012)

#### Taxon discussion

See general Discussion below.

## Analysis

**Figure 1.**
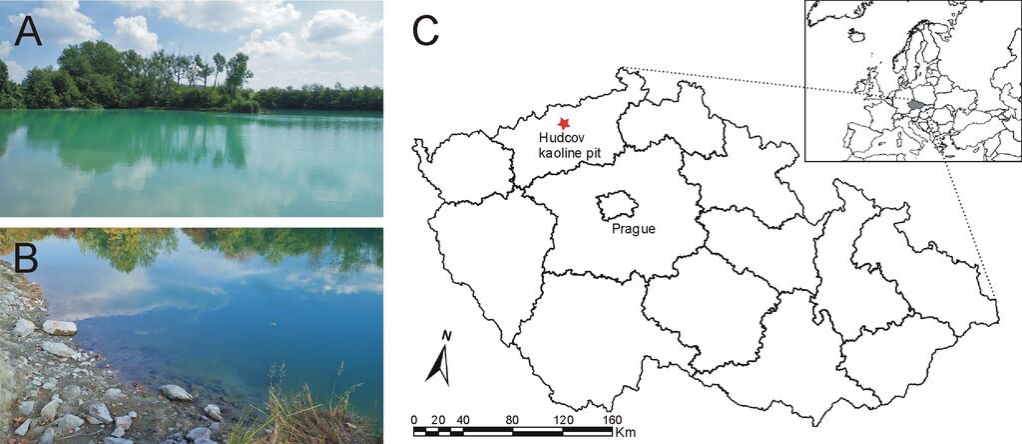
Overview on the locality Hudcov kaoline pit (A), focused view on one of the sampled sites (B) and map of the Czech Republic with marked locality where *Ephemeraglaucops* was recorded (C).

A total of 27 larvae of *E.glaucops* were collected in a single locality, a kaolin pit near the village of Hudcov (Fig. 1, locality code: 5348d; the code corresponds with the number of the Czech faunistic grid mapping system), 50°37'14.67"N, 13°46'30.12"E, 217 m a.s.l., 11–14.vii. 2018 (24 larvae), 21–22.ix.2018, (3 larvae). All sampling sites were located at a mean distance of 65 cm from the shore at a depth of 20.5 cm. The kaolin pit is located in the agricultural landscape, surrounded by crop fields, partly meadows and forest. The substrate was a mixture of kaolin clay, stones, sand and organic silt, mostly with open water or very sparse vegetation (*Phragmitesaustralis*, roots of the terrestrial vegetation). The measured mean values of physico-chemical parameters in the sites, where *E.glaucops* was found, were as follows: temperature 21.6°C, conductivity 1000.5 µS.cm^-1^, pH 8.58, dissolved oxygen 9.47 mg.l^-1^, substrate pH 6.7 and water transparency 37.7 cm. Fish, namely native *Anguillaanguilla*, *Rutilusrutilus*, *Cyprinuscarpio* and non-native *Pseurodasboraparva*, were observed at the locality (Kolar et al., unpublished). Most individuals (26) were sampled with a boxtrap and only one individual was collected by sweeping with a strainer.

The material was preserved in denaturised ethanol (EtOH) and identified using the keys of Bauernfeind and Humpesch (2001) and Eiseler (2005). Photographs were taken using a Canon EOS1200D camera with a macro lens Canon MP-E 65 mm, attached to WeMacro Rail. All photographs were then stacked in Helicon Focus 6.3 and enhanced with Adobe Photoshop CS5. The material is housed in the collection of the Biology Centre CAS, Institute of Entomology (IECA).

## Discussion

### Species identification

**Figure 2.**
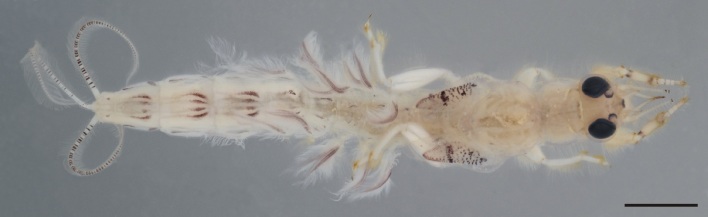
Habitus of *Ephemeraglaucops*. Scale: 2 mm.

From all three remaining species of *Ephemera* distributed in the Czech Republic, the larvae of *E.glaucops* can be distinguished by the colouration of the abdomen (terga VII–IX with two pairs of relatively narrow stripes, median pair less pronounced and terga II–VI with one pair of indistinct dark markings, Fig. 2). The shape of the fore-tibiae with apical protuberance (Fig. 3c) is also characteristic. However, this protuberance is sometimes reported as poorly developed and more easily observable from the dorsal view, since it bends slightly outwards (Wagner, pers. comm.). The number of prominent setae on pedicel is given as 2–3 (Bauernfeind and Humpesch 2001, Bauernfeind and Soldán 2012), which corresponds with our specimens. Apart from the colouration and shape of the fore-tibiae, this character can be used to distinguish the species from co-occurring *E.vulgata* with 4–9 setae (Bauernfeind and Humpesch 2001). In our samples from northern Bohemia, *E.glaucops* is also distinguishable from *E.vulgata* by smaller size (the last-instar larvae 13.5–14.5 mm long in *E.glaucops*, compared to 20–21 mm in *E.vulgata*). In our specimens of *E.glaucops*, we also noted a characteristic shape of clypeus, with strongly divergent lateral margins (Fig. 3a). This character was not used in recent identification keys and might be a subject of variability.

### Habitat properties and species distribution

Of the total 20 post-industrial freshwater localities investigated in the area of northern and eastern Bohemia, *E.glaucops* was recorded only in the locality "kaolin pit Hudcov". Other mayfly species co-occurring in the same locality include related burrowing Ephemera (Ephemera) vulgata Linnaeus, 1758, walking/sprawling species *Caenisluctuosa* (Burmeister, 1839) and *Caenishoraria* (Linnaeus, 1758) and fish-like active species Cloeon (Cloeon) dipterum (Linnaeus, 1761) s.l. and Cloeon (Similicloeon) simile Eaton, 1870 s.l.; they are all very common in the Czech mayfly fauna (Zahrádková et al. 2009).

Overall scarce historical records of *E.glaucops* from the Czech Republic were limited to lowland rivers in north-west Bohemia: Ohře (Eger) River near Cheb (Dalla-Torre 1878), Berounka River in Nová Huť (coll. Klapálek, National Museum, Prague, R. Godunko rev.) and Labe (Elbe) River near Ústí nad Labem (Pawlik 1933). The finding of the species in the kaolin pit is consistent with numerous recent records from Germany, The Netherlands and Austria, documenting the colonisation of artificial habitats, such as lakes and ponds in open-pit lignite mines and sand and gravel pits (Jacob et al. 1975, Braasch and Mey 1977, Haybach and Fischer 1994, Hutter and Graf 1994, Zahn 2003). These habitats were often in the early successional stage, without macrophytes and with high water transparency (Haybach and Fischer 1994, Hutter and Graf 1994, Zahn 2003). Larvae dwelled in shallow gravel-sand littoral zone, freely burrowing in the sediment (without burrowing of tubes) (Haybach and Fischer 1994). It seems larvae colonise only littorals with stable substrate, for example, places where swimming is prohibited in lakes (Haybach and Fischer 1994). Assuming from the presence of *E.glaucops* in just a single locality, it is still very rare in the Czech Republic. The species was not found in recent studies examining a similar geographic area in northern Bohemia (Polášková et al. 2017, Bartošová et al. 2019, Poláková et al. 2022), nor in other localities we sampled in 2018–2019 (Kolar et al., unpublished). However, *E.glaucops* is likely recovering in some areas, as an increase in abundance of the species has recently been reported from Germany (Haybach 2021) and new records have been published from Hungary (Kovács 2001), Belgium (Lock and Goethals 2011) and Croatia (Vilenica et al. 2019). The closest point of occurrence to our locality near Hudcov was reported by Lässig et al. (2000) in Weiditz (north from Rochlitz, Saxony, Germany), approximately 85 km from our sampling site. Thanks to the high dispersal ability of the species (cf. Blanke et al. 1993), the spreading of species in northern Bohemia is likely. For monitoring the species occurrence, the usage of light trapping to collect adults would be very useful to employ in the future, since it generally represents an effective way for collecting *E.glaucops*.

Our study shows the remarkable value of post-industrial sites for aquatic biodiversity, which is well-known for terrestrial biota (Tropek and Řehounek 2011, Tropek et al. 2012, Tropek et al. 2013, Heneberg and Řezáč 2014). Many recent studies (Tichanek and Tropek 2015, Harabiš 2016, Sroka et al. 2016, Polášková et al. 2017, Bartošová et al. 2019, Kolar et al. 2021a, Kolar et al. 2021b, Poláková et al. 2022) recorded numerous aquatic species from different groups, mainly aquatic beetles, heteropterans, dipterans (especially Stratiomyidae, Psychodidae, Limoniidae and Chironomidae) and odonates, which found suitable conditions in these man-made habitats, probably due to the degradation of natural wetlands. However, the biota of kaolin pits is still unknown with the exception of dragonflies and aquatic beetles (Boukal et al. 2007, Bobrek 2020). On the other hand, these localities could also serve as ecological traps, where the species cannot survive due to different stressors, such as unpredictable disturbances due to soil instability or extreme ion concentrations (Harabiš and Dolný 2012, Bartošová et al. 2019, Chmelová et al. 2021).

As an effective means of management to increase the abundances of *E.glaucops*, we recommend regular small-scale disturbances of shallow parts of the waterbody to slow down succession, especially by removing macrophyte vegetation as *E.glaucops* prefers the early successional stage. On the other hand, the disturbances should be applied in a mosaic i.e. with parts left overgrown, as different groups of organisms could have different habitat requirements (e.g. Sroka et al. 2016, Kolar et al. 2021a, Kolar et al. 2021b).

## Supplementary Material

XML Treatment for Ephemera (Sinephemera) glaucops

## Figures and Tables

**Figure 3a.**
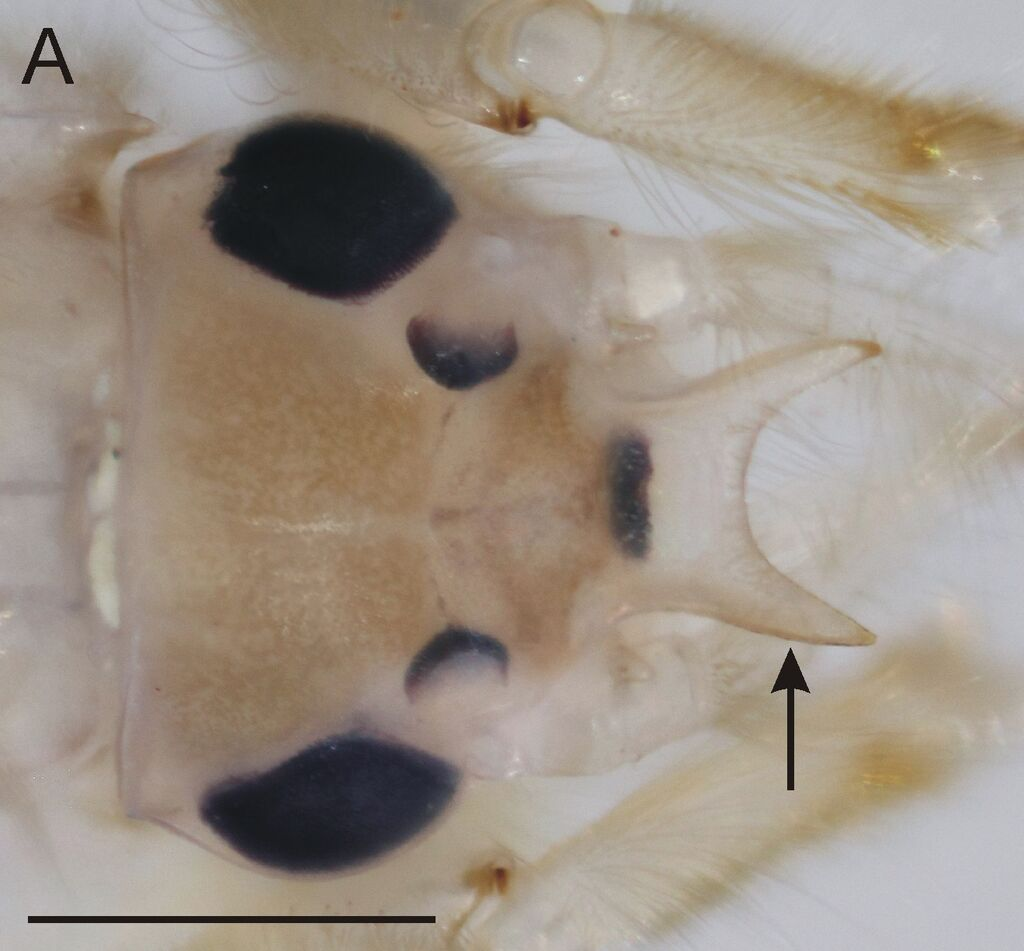
Head of *E.glaucops*, dorsal view (arrow points to clypeus).

**Figure 3b.**
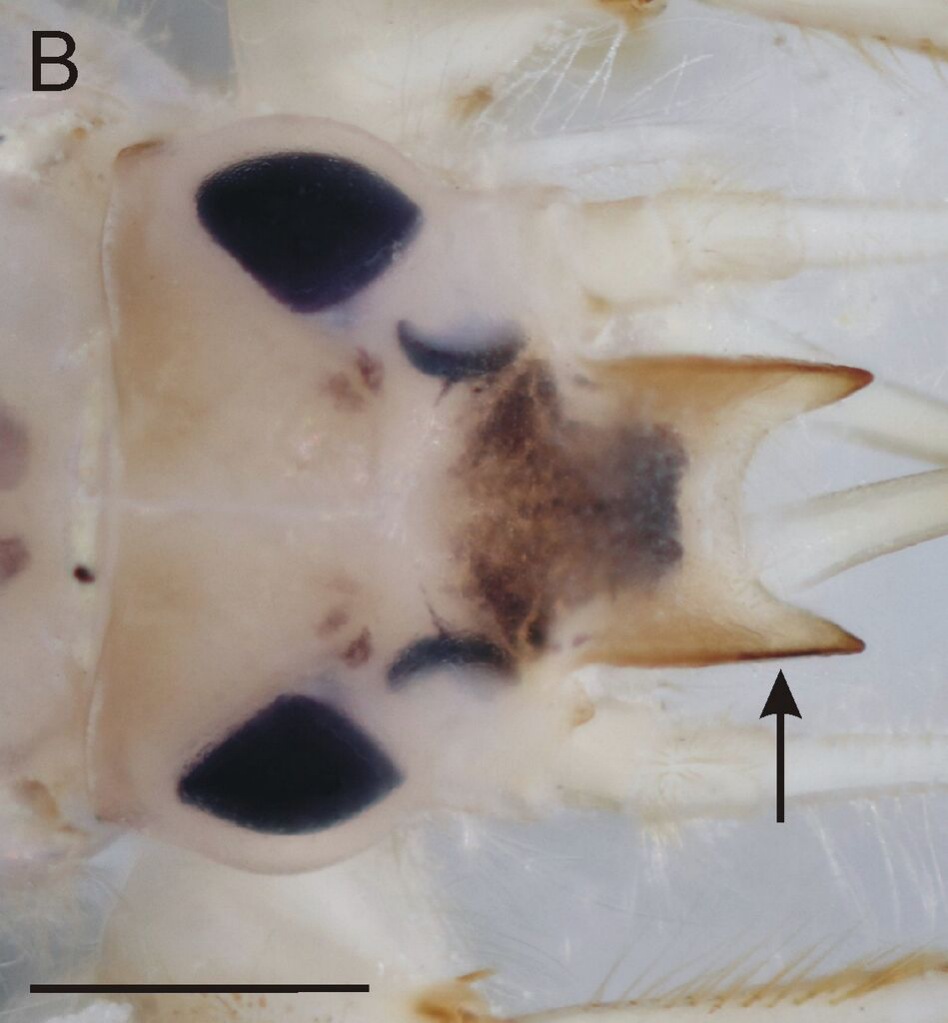
Head of *E.vulgata*, dorsal view (arrow points to clypeus).

**Figure 3c.**
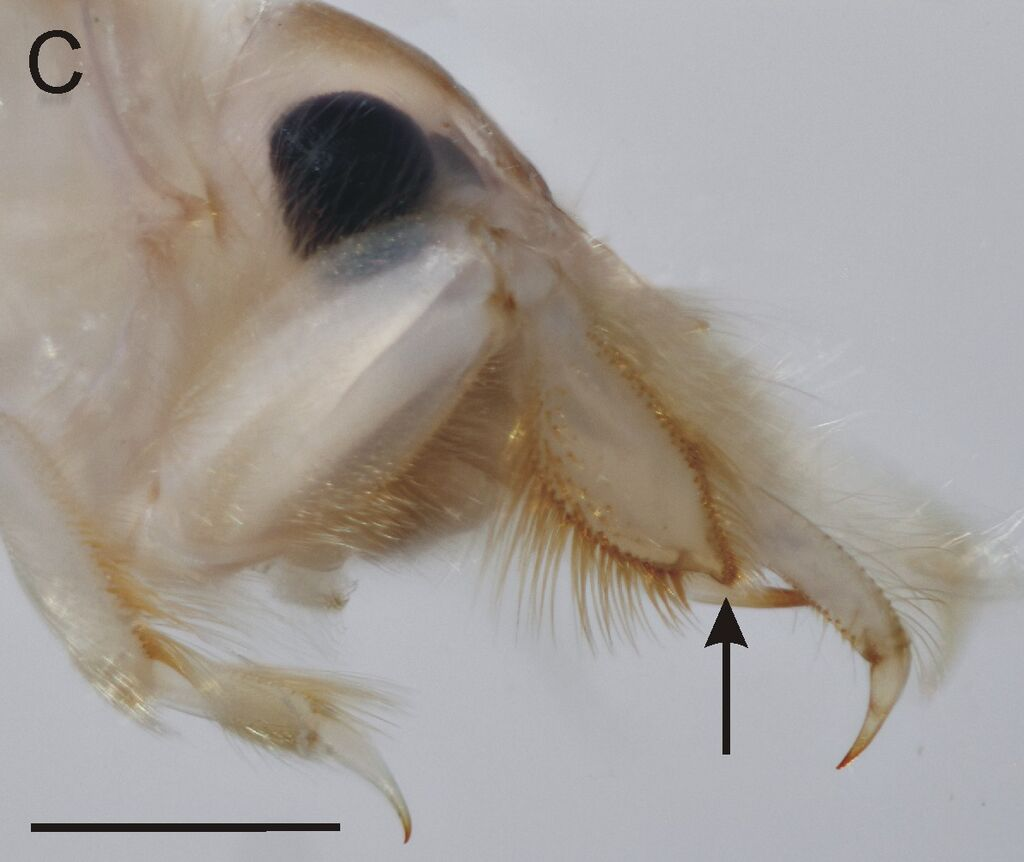
Fore-leg of *E.glaucops*, lateral view (arrow points to apical protuberance on fore-tibia).

**Figure 3d.**
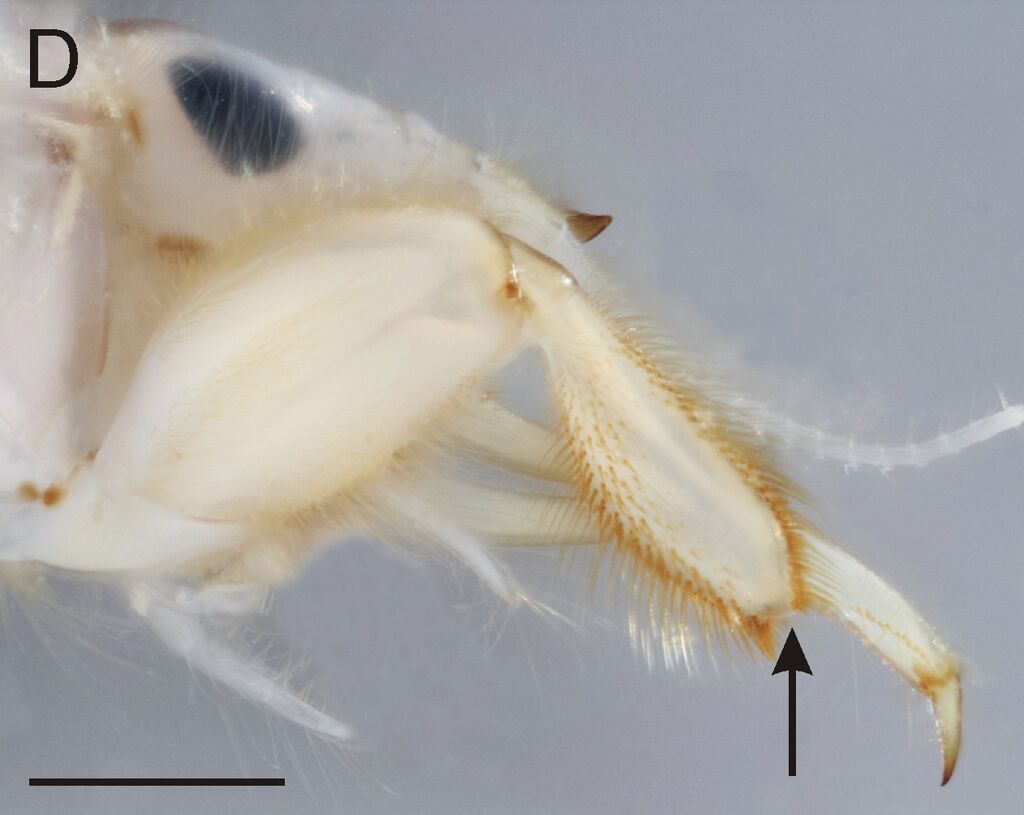
Fore-leg of *E.vulgata*, lateral view (arrow points to apex of fore-tibia without protuberance).

## References

[B7911492] Bartošová M., Schenková J., Polášková V., Bojková J., Šorfová V., Horsák M. (2019). Macroinvertebrate assemblages of the post-mining calcareous stream habitats: Are they similar to those inhabiting the natural calcareous springs?. Ecological Engineering.

[B7911503] Bauernfeind E., Humpesch U. H. (2001). Die Eintagsfliegen Zentraleuropas – Bestimmung und Ökologie.

[B7911511] Bauernfeind E., Soldán T. (2012). The Mayflies of Europe.

[B7919359] Blanke D., Dörfer K., Böwingloh F. (1993). Wiederfund von *Ephemeraglaucops* PICTET, 1843 für Niedersachsen (Insecta: Ephemeroptera). Braunschweiger Naturkundliche Schriften.

[B7911519] Bobrek R. (2020). High biodiversity in a city centre: Odonatofauna in an abandoned limestone quarry. European Journal of Environmental Sciences.

[B7911528] Boukal D. S., Boukal M., Fikáček M., Hájek J., Klečka J., Skalický S., Šťastný J., Dušan T. (2007). Catalogue of water beetles of the Czech Republic (Coleoptera: Sphaeriusidae, Gyrinidae, Haliplidae, Noteridae, Hygrobiidae, Dytiscidae, Helophoridae, Georissidae, Hydrochidae, Spercheidae, Hydrophilidae, Hydraenidae, Scirtidae, Elmidae, Dryopidae, Limnich). Klapalekiana.

[B7911541] Braasch D., Mey W. (1977). Ein weiterer Fund von *Ephemeraglaucops* Pictet (Ephemeroptera) in der DDR. Entomologische Nachrichten.

[B7911550] Chmelová E., Kolar V., Jan J., Carreira B. M., Landeira-Dabarca A., Otáhalová Š., Poláková M., Vebrová L., Borovec J., Boukal D. S., Tropek R. (2021). Valuable secondary habitats or hazardous ecological traps? Environmental risk assessment of minor and trace elements in fly ash deposits across the Czech Republic. Sustainability.

[B7911586] Dalla-Torre C. G. (1878). Entomologische Notizen aus dem Egerlande. Lotos.

[B7911642] Eiseler B. (2005). Bildbestimmungsschlüssel für die Eintagsfliegenlarven der deutschen Mittelgebirge und des Tieflandes. Lauterbornia.

[B7911711] Harabiš F., Dolný A. (2012). Human altered ecosystems: suitable habitats as well as ecological traps for dragonflies (Odonata): the matter of scale. Journal of Insect Conservation.

[B7911702] Harabiš F. (2016). High diversity of odonates in post-mining areas: Meta-analysis uncovers potential pitfalls associated with the formation and management of valuable habitats. Ecological Engineering.

[B7930827] Haybach A., Fischer J. (1994). Zur Kenntnis der Eintagsfliegenfauna (Insecta: Ephemeroptera) von Rheinland-Pfalz. Lauterbornia.

[B7911720] Haybach A., Ries M. (2021). Rote Liste gefährdeter Tiere, Pflanzen und Pilze Deutschlands, Band 5: Wirbellose Tiere (Teil 3).

[B7915969] Heneberg P., Řezáč M. (2014). Dry sandpits and gravel-sandpits serve as key refuges for endangered epigeic spiders (Araneae) and harvestmen (Opiliones) of Central European steppes aeolian sands. Ecological Engineering.

[B7915978] Hutter G., Graf W. (1994). Wiederfund von *Ephemeraglaucops* Pictet 1843-1845 in Österreich. Lauterbornia.

[B7915987] Jacob U., Kauk S., Klima F. (1975). Eine ephemeropterologische Überraschung – *Ephemeraglaucops* Pictet bei Leipzig. Entomologische Nachrichten.

[B7916005] Klečka J., Boukal D. S. (2014). Lazy ecologist's guide to water beetle diversity: Which sampling methods are the best?. Ecological Indicators.

[B7969500] Kluge N. J. (2004). The Phylogenetic System of Ephemeroptera.

[B7916014] Koese B., Kalkman V. J. (2008). De soorten van het leefgebiedenbeleid.

[B7916063] Kolar V., Tichanek F., Tropek R. (2021). Evidence-based restoration of freshwater biodiversity after mining: Experience from Central European spoil heaps. Journal of Applied Ecology.

[B7916072] Kolar V., Vlašánek P., Boukal D. S. (2021). The influence of successional stage on local odonate communities in man-made standing waters. Ecological Engineering.

[B7916081] Kovács T. (2001). Somogy megye kérészeinek katalógusa (Ephemeroptera). Natura Somogyiensis.

[B7916090] Lässig A., Brockhaus T., Küttner R. (2000). Einige interessante Insektennachweise aus dem Raum Rochlitz und Colditz (Lepidoptera, Odonata, Ephemeroptera, Trichoptera). Entomologische Nachrichten und Berichte.

[B7915996] Lock K., Goethals P. L. M. (2011). Distribution and ecology of the mayflies (Ephemeroptera) of Flanders (Belgium). Annales de Limnologie.

[B7918148] Malmquist B., Rundle S. (2002). Threats to the running water ecosystems of the world. Environmental Conservation.

[B7918313] Pawlik E. (1933). Einstagfliegen aus dem Elbetale bei Aussig. Natur und Heimat.

[B7918322] Poláková M., Straka M., Polášek M., Němejcová D. (2022). Unexplored freshwater communities in post-mining ponds: effect of different restoration approaches. Restoration Ecology.

[B7918340] Polášková V., Schenková J., Bartošová M., Rádková V., Horsák M. (2017). Post-mining calcareous seepages as surrogate habitats for aquatic macroinvertebrate biota of vanishing calcareous spring fens. Ecological Engineering.

[B8103093] Sartori M., Bauernfeind E. (2020). Mayfly types and additional material (Insecta: Ephemeroptera) examined by F.-J. Pictet and A.-E. Pictet, housed in the Museums of Natural History of Geneva and Vienna. Revue Suisse de Zoologie.

[B7918360] Soldán T., Zahrádková S., Helešic J., Dušek L., Landa V. (1998). Distributional and quantitative patterns of Ephemeroptera and Plecoptera in the Czech Republic: a possibility of detection of long-term changes of aquatic biotopes. Folia Facultatis Scientiarum Naturalium Universitatis Masarykianae Brunensis.

[B7918387] Soldán T., Bojková J., Zahrádková S., Hejda R., Farkač J., Chobot K. (2017). Red List of threatened species of the Czech Republic. Invertebrates.

[B7918409] Sroka P., Klecka J., Boukal D. S. (2016). Spatial heterogeneity and habitat permanence affect community assembly, structure and phenology of mayflies (Ephemeroptera) in sandpit pools. Zoosymposia.

[B7918419] Studemann D., Landolt P., Sartori M., Hefti D., Tomka I. (1992). Ephemeroptera.

[B7918428] Tichanek F., Tropek R. (2015). Conservation value of post-mining headwaters: Drainage channels at a lignite spoil heap harbour threatened stream dragonflies. Journal of Insect Conservation.

[B7930845] Tropek R., Řehounek J. (2011). Bezobratlí postindustriálních stanovišť: Význam, ochrana a management.

[B7918977] Tropek R., Kadlec T., Hejda M., Kocarek P., Skuhrovec J., Malenovsky I., Vodka S., Spitzer L., Banar P., Konvicka M. (2012). Technical reclamations are wasting the conservation potential of post-mining sites. A case study of black coal spoil dumps. Ecological Engineering.

[B7919031] Tropek R., Cerna I., Straka J., Cizek O., Konvicka M. (2013). Is coal combustion the last chance for vanishing insects of inland drift sand dunes in Europe?. Biological Conservation.

[B7919041] Vilenica M., Vučković N., Mihaljević Z. (2019). Littoral mayfly assemblages in South-East European man-made lakes. Journal of Limnology.

[B7919726] Zahn S. (2003). Nachweise der Eintagsfliege *Ephemeraglaucops* (Insecta: Ephemeroptera; Ephemeridae) in Bergbaurestgewässern Brandenburgs und Sachsens (Deutschland). Lauterbornia.

[B7919050] Zahrádková S., Soldán T., Bojková J., Helešic J., Janovská H., Sroka P. (2009). Distribution and biology of mayflies (Ephemeroptera) of the Czech Republic: present status and perspectives. Aquatic Insects.

